# Gene-based analysis of ADHD using PASCAL: a biological insight into the novel associated genes

**DOI:** 10.1186/s12920-019-0593-5

**Published:** 2019-10-24

**Authors:** Aitana Alonso-Gonzalez, Manuel Calaza, Cristina Rodriguez-Fontenla, Angel Carracedo

**Affiliations:** 1 Grupo de Medicina Xenómica, Fundación Instituto de Investigación Sanitaria de Santiago de Compostela (FIDIS), Center for Research in Molecular Medicine and Chronic Diseases (CIMUS), Universidad de Santiago de Compostela, Santiago de Compostela, Spain; 2 Grupo de Medicina Genómica, CIBERER, CIMUS (Centre for Research in Molecular Medicine and Chronic Diseases), Universidade de Santiago de Compostela, Santiago de Compostela, Spain

**Keywords:** ADHD (attention-deficit hyperactivity disorder), GBA (gene-based analysis), GWAS (genome wide association study), NDDs (neurodevelopmental disorders), PGC (Psychiatric Genomics Consortium), DEG (differentially expressed gene) analysis, Gene-network analysis, PASCAL (pathway scoring algorithm)

## Abstract

**Background:**

Attention-Deficit Hyperactivity Disorder (ADHD) is a complex neurodevelopmental disorder (NDD) which may significantly impact on the affected individual’s life. *ADHD* is acknowledged to have a high *heritability* component (70–80%). Recently, a meta-analysis of GWAS (Genome Wide Association Studies) has demonstrated the association of several independent loci. Our main aim here, is to apply PASCAL (pathway scoring algorithm), a new gene-based analysis (GBA) method, to the summary statistics obtained in this meta-analysis. PASCAL will take into account the linkage disequilibrium (LD) across genomic regions in a different way than the most commonly employed GBA methods (MAGMA or VEGAS (Versatile Gene-based Association Study)). In addition to PASCAL analysis a gene network and an enrichment analysis for KEGG and GO terms were carried out. Moreover, GENE2FUNC tool was employed to create gene expression heatmaps and to carry out a (DEG) (Differentially Expressed Gene) analysis using GTEX v7 and BrainSpan data.

**Results:**

PASCAL results have revealed the association of new loci with ADHD and it has also highlighted other genes previously reported by MAGMA analysis. PASCAL was able to discover new associations at a gene level for ADHD: *FEZF1 (p*-value: 2.2 × 10^− 7^) and *FEZF1-AS1* (*p*-value: 4.58 × 10^− 7^). In addition, PASCAL has been able to highlight association of other genes that share the same LD block with some previously reported ADHD susceptibility genes. Gene network analysis has revealed several interactors with the associated ADHD genes and different GO and KEGG terms have been associated. In addition, GENE2FUNC has demonstrated the existence of several up and down regulated expression clusters when the associated genes and their interactors were considered.

**Conclusions:**

PASCAL has been revealed as an efficient tool to extract additional information from previous GWAS using their summary statistics. This study has identified novel ADHD associated genes that were not previously reported when other GBA methods were employed. Moreover, a biological insight into the biological function of the ADHD associated genes across brain regions and neurodevelopmental stages is provided.

## Background

Attention-deficit/Hyperactivity Disorder (ADHD) is a common neurodevelopmental disorder (NDD) characterized by an ongoing pattern of inattention and/or hyperactivity that directly interferes with social functioning [1]. The worldwide estimated prevalence of ADHD is about 5% in children and adolescents and about 2.5% in adult population [2].

ADHD is a complex neurodevelopmental disorder, meaning that both environmental and genetic factors are involved in its etiology. However, the genetics basis of ADHD remains largely unknown due to its clinical heterogeneity. Thus, ADHD presents comorbidity with other psychiatric and neurodevelopmental disorders such as schizophrenia, depression, bipolar disorder and autism spectrum disorder (ASD) [2]. The high heritability of ADHD (70–80%) was demonstrated by family and twin studies. Therefore, different genetic approaches were employed to search for ADHD susceptibility genes [3, 4]. Polygenic liability models has pointed towards a model in which both, single-nucleotide polymorphisms (SNPs) and rare copy number variants (CNVs) are involved in ADHD genetics For these reasons, it is considered that common variation. explains a substantial fraction of ADHD heritability [5–7]. However, early ADHD (GWAS) have failed to detect robust signals surpassing the established significance threshold (5 × 10 ^− 8^). This could be possibly due to the lack of standardized phenotyping protocols and the need of a larger number of cases and controls that allow the detection of common variants with an small effect [8]. Although none of the findings from these early GWAS were genome-wide significant, some interesting loci were highlighted: *CDH13, SLC9A9, NOS1* and *CNR1* [9].

The latest GWAS meta-analyses conducted by the Psychiatric Genomic Consortium (PGC) have increased the sample size up to ten thousands of cases and controls after a rigorous phenotypic characterization. Thus, this study has identified 12 independent loci carrying 304 SNPs that surpasses the required threshold for genome-wide significance. Some of the main associated SNPs are located within a large gene cluster located on chromosome 1 *(ST3GAL3, KDM4A, KDM4A-AS1, PTPRF, SLC6A9, ARTN, DPH2, ATP6V0B, B4GALT2, CCDC24, IPO13), and SPAG16, FOXP2, PCDH7, SORCS3, DUSP5* *SEMA6D* [10].

Gene-based analysis (GBA) strategies are additional analyses focused on the study of genes as testing units with a biological entity. GBA generally employs GWAS summary statistics without the need of individual genotypes. Thus, it takes into account all SNPs within a gene and the correlations among them to construct a statistic for each single gene [11]. The association results at a gene-level are useful to carry out secondary approaches and to characterize their biological functions [12]. GBA for ADHD has been already performed by MAGMA, one of the most commonly employed approaches together with VEGAS (Versatile Gene-based Association Study) [13, 14]*.* Several genes have shown significant association with ADHD after MAGMA analysis: *ST3GAL3, KDM4A, PTPRF, SZT2, TIE1, MPL, CDC20, HYI, SLC6A9, ELOVL1, CCDC24* (chromosome 1); *MANBA* (chromosome 4); *MEF2C 5* (chromosome 5); *FOXP2* (chromosome 7); *SORCS3, CUBN (*chromosome 10); *DUSP6* (chromosome 12); *SEMA6D* (chromosome 15); *CDH8* (chromosome 16).

However, it was recently released a novel GBA strategy called PASCAL (Pathway Scoring Algorithm) PASCAL allows to generate gene scores by aggregating SNP *p*-values from GWAS meta-analysis while correcting for linkage disequilibrium (LD) structure. Moreover, PASCAL corrects for multiple testing while adjusting individual *p*-values depending on the correlation among SNPs. Thus, the construction of the correlation matrix is one of the main differences in comparison with other GBA methods as MAGMA. MAGMA also creates a SNP matrix based on principal components but it eliminates those SNPs that contribute with small eigen values [15]*.*

Therefore, the main aim of this paper is to apply PASCAL to several public ADHD GWAS meta-analysis data (whole European, females and males). It is expected that PASCAL will help to identify new associations at a gene-level as well as to redefine those previously found by MAGMA. In addition, the list of ADHD associated genes will be studied through gene-network and functional annotation approaches in order to gain information about their potential biological role.

## Results

### Gene-based-analysis

**Fig. 1:**
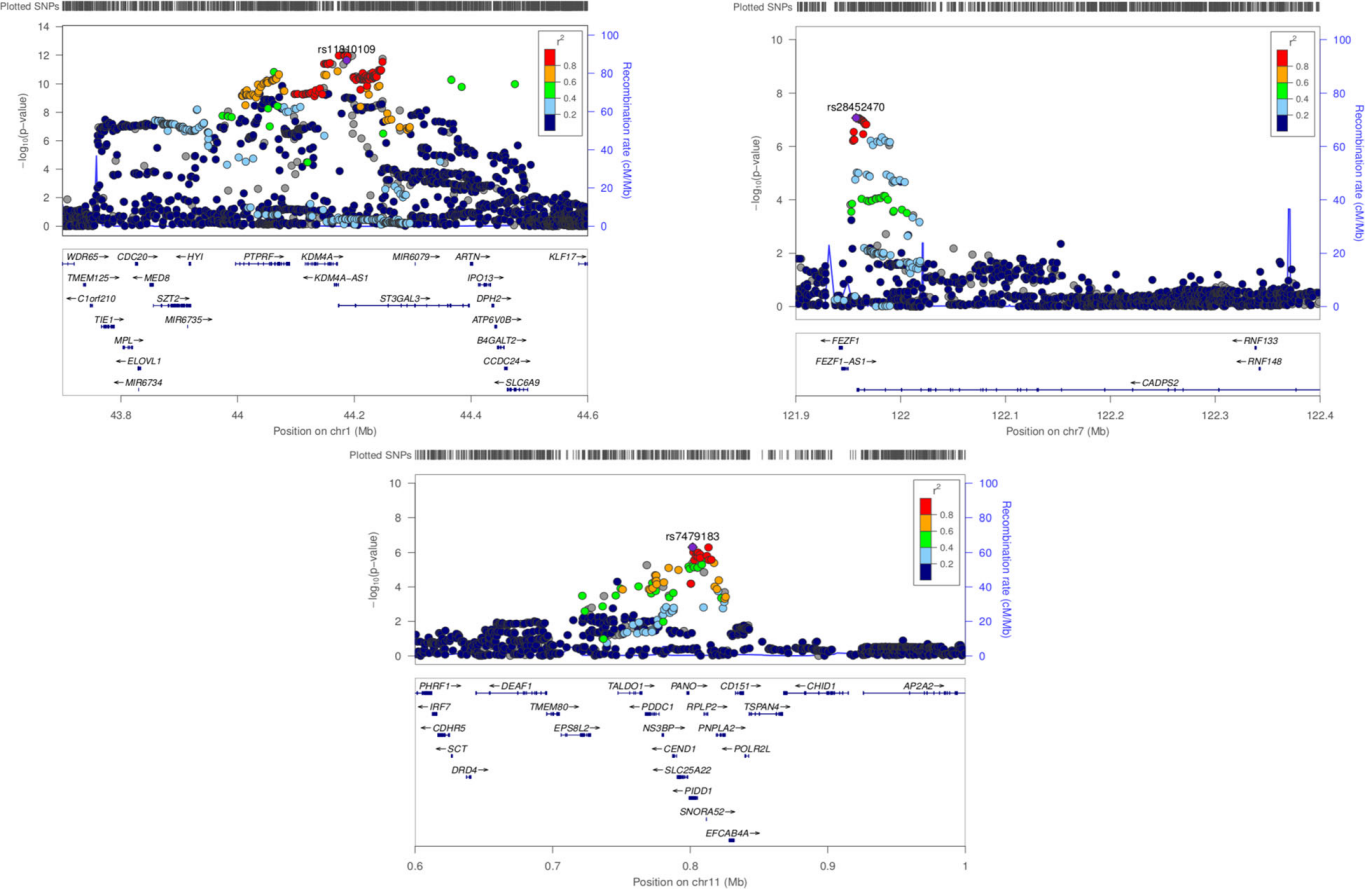
Regional association plots for ADHD GWAS meta-analysis (chromosomes 1, 7 and 11). PASCAL has revealed novel associations at a gene level for *MED8* (chr1), *FEZF1* and *FEZF1-AS1* (chr 7) and *NS3BP* and *PDDC1* (chr11). Regional plot for chromosome 1 has been constructed with rs11810109 as index SNP (*r*^*2*^ = 0.99 with the lead SNP, rs11420276) due to the lack of LD data for this marker

GBA of ADHD done with summary statistics from European meta-analysis has revealed association of 19 loci surpassing the required Bonferroni threshold (2.26 × 10^− 6^). These loci are located on chromosomes 1, 7, 10, 11, 15 and 16. *MED8,* was highlighted as associated by PASCAL in comparison with results obtained by MAGMA (Table 1) (Fig. 1). Moreover, other genes were associated in chromosome 7 (*FEZF1, FEZF1-AS1*) and chromosome 11 (*NS3BP, PDDC1).* Regional plot around *FEZF1* has revealed an interesting and well-delimited genomic region located between two linkage peaks and encompassing the 3’UTR of *CADPS2* gene (Fig. 1).

**Table 1:** Genes reported by PASCAL for ADHD that surpass the established Bonferroni threshold (*p*-value < 2.26 × 10^− 6^). Columns shown gene, chromosome, start and end position for each gene, the number of SNPs included in the analysis for each gene and the corresponding *p* -value given by PASCAL

Gene	chromosome	start position	end position	number of SNPs	PASCAL *p*-value
*KDM4A*	chr1	44115796	44171189	287	1 × 10^−12^
*KDM4A-AS1*	chr1	44165355	44173012	162	1.46 × 10^−11^
*ST3GAL3*	chr1	44171494	44396837	538	6.72 × 10^−11^
*PTPRF*	chr1	43996546	44089343	386	1.81 × 10^−10^
*SZT2*	chr1	43855555	43919918	147	1.51 × 10^− 8^
*DUSP6*	chr12	89741836	89746296	169	1.56 × 10^− 8^
*HYI*	chr1	43916673	43919660	91	2.37 × 10^− 8^
*CDC20*	chr1	43824625	43828873	92	2.51 × 10^− 8^
*ELOVL1*	chr1	43829067	43833745	94	2.52 × 10^− 8^
***MED8***	chr1	43849578	43855483	96	2.99 × 10^− 8^
***MPL***	chr1	43803474	43820135	111	3.14 × 10^− 8^
*TIE1*	chr1	43766565	43791552	127	8.84 × 10^− 8^
***FEZF1***	chr7	121941447	121944565	200	2.2 × 10^− 7^
*SORCS3*	chr10	106400858	107024993	1773	3.66 × 10^− 7^
***FEZF1-AS1***	chr7	121943711	121950131	217	4.58 × 10^−7^
*CDH8*	chr16	61685914	62070739	815	9.27 × 10^− 7^
*SEMA6D*	chr15	47476402	48066420	1394	1.4 × 10^−6^
***NS3BP***	chr11	779453	780755	180	1.81 × 10^− 6^
***PDDC1***	chr11	767222	777487	194	1.91 × 10^− 6^

Boldface entries remark those genes highlighted by PASCAL and not by MAGMA

**Fig. 2:**
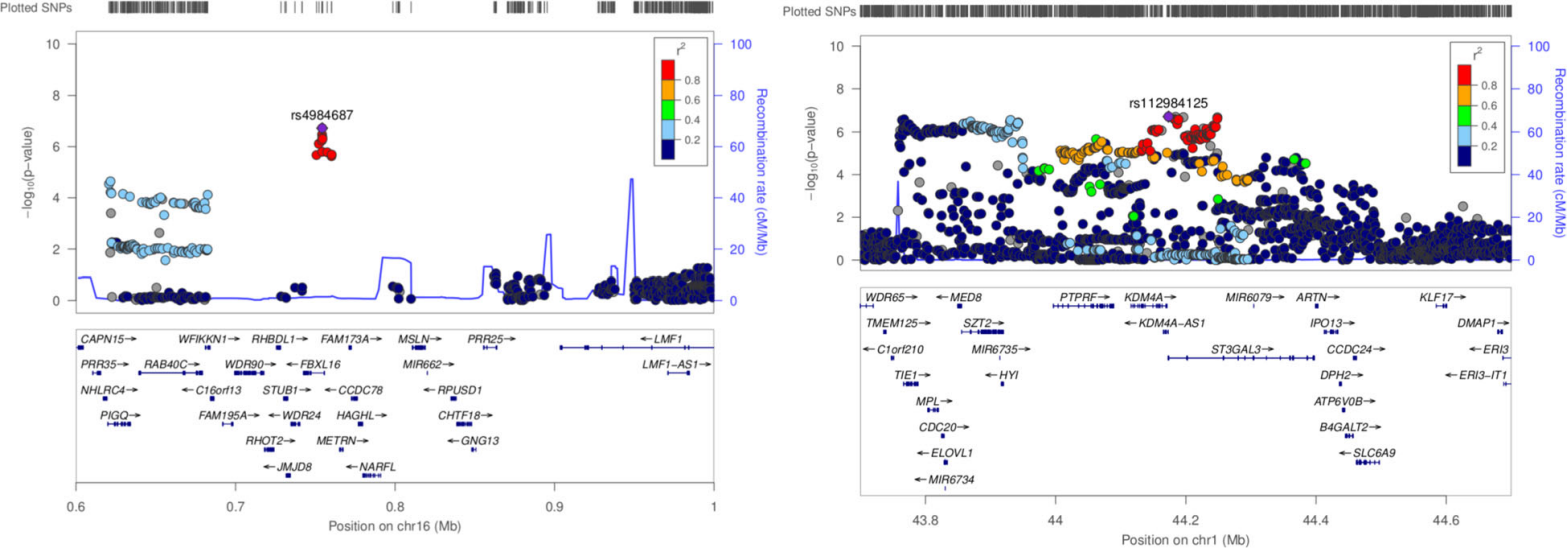
Regional association plots for ADHD GWAS meta-analysis; females (chromosome 16) and males (chromosome 1). Both regions contain PASCAL associated genes

GBA of the female subgroup meta-analysis has also revealed interesting results. Therefore, 9 new loci located on chromosome 16 were associated: *JMJD8, NARFL, WDR24, FBXL16, METRN, FAM173A, CCDC78, HAGHL* and *STUB1* (Table 2) (Fig. 2).

**Table 2:** Genes reported by PASCAL for ADHD females that surpass the established Bonferroni threshold (*p*-value < 2.26 × 10^− 6^). Columns shown gene, chromosome, start and end position for each gene, the number of SNPs included in the analysis for each gene and the corresponding *p*-value given by PASCAL

Gene	chromosome	start position	end position	number of SNPs	PASCAL *p*-value
*JMJD8*	chr16	731666	734439	23	9.18 × 10^− 7^
*NARFL*	chr16	779768	790997	21	1.12 × 10^− 6^
*WDR24*	chr16	734701	740400	17	1.26 × 10^− 6^
*FBXL16*	chr16	742499	755825	21	1.32 × 10^− 6^
*METRN*	chr16	765172	767480	22	1.39 × 10^−6^
*FAM173A*	chr16	771141	772590	22	1.39 × 10^−6^
*CCDC78*	chr16	772581	776880	22	1.39 × 10^−6^
*HAGHL*	chr16	776957	779715	22	1.39 × 10^−6^
*STUB1*	chr16	730114	732768	30	1.55 × 10^−6^

GBA in the male subset has not revealed any novel association at a gene level and the associated loci were also identified when the whole European population analysis (cluster in chromosome 1) was carried out. However, lower association levels were reported in males (Table 3) (Fig. 2). Moreover, ADHD GBA done in the male subgroup highlights different top associated genes (*STZ2, ELOVL1, CDC20*) in comparison with those found in the whole European population (*KDM4A, KDM4A-AS1, ST3GAL3)* (Tables 1 and 3).

**Table 3:** Genes reported by PASCAL for ADHD males that surpass the established Bonferroni threshold (*p*-value < 2.26 × 10^− 6^). Columns shown gene, chromosome, start and end position for each gene,the number of SNPs included in the analysis for each gene and the corresponding *p* value given by PASCAL

Gene	chromosome	start position	end position	number of SNPs	PASCAL *p*-value
*SZT2*	chr1	43,855,555	43,919,918	142	3.05 × 10^−7^
*ELOVL1*	chr1	43,829,067	43,833,745	91	3.62 × 10^− 7^
*CDC20*	chr1	43,824,625	43,828,873	89	3.64 × 10^−7^
*MPL*	chr1	43,803,474	43,820,135	102	3.77 × 10^−7^
*MED8*	chr1	43,849,578	43,855,483	94	3.9 × 10^−7^
*HYI*	chr1	43,916,673	43,919,660	88	4.83 × 10^−7^
*TIE1*	chr1	43,766,565	43,791,552	116	7.21 × 10^−7^
*KDM4A*	chr1	44,115,796	44,171,189	281	8.09 × 10^−7^
*KDM4A-AS1*	chr1	44,165,355	44,173,012	160	1.82 × 10^−6^

### Gene network analysis

**Fig. 3:**
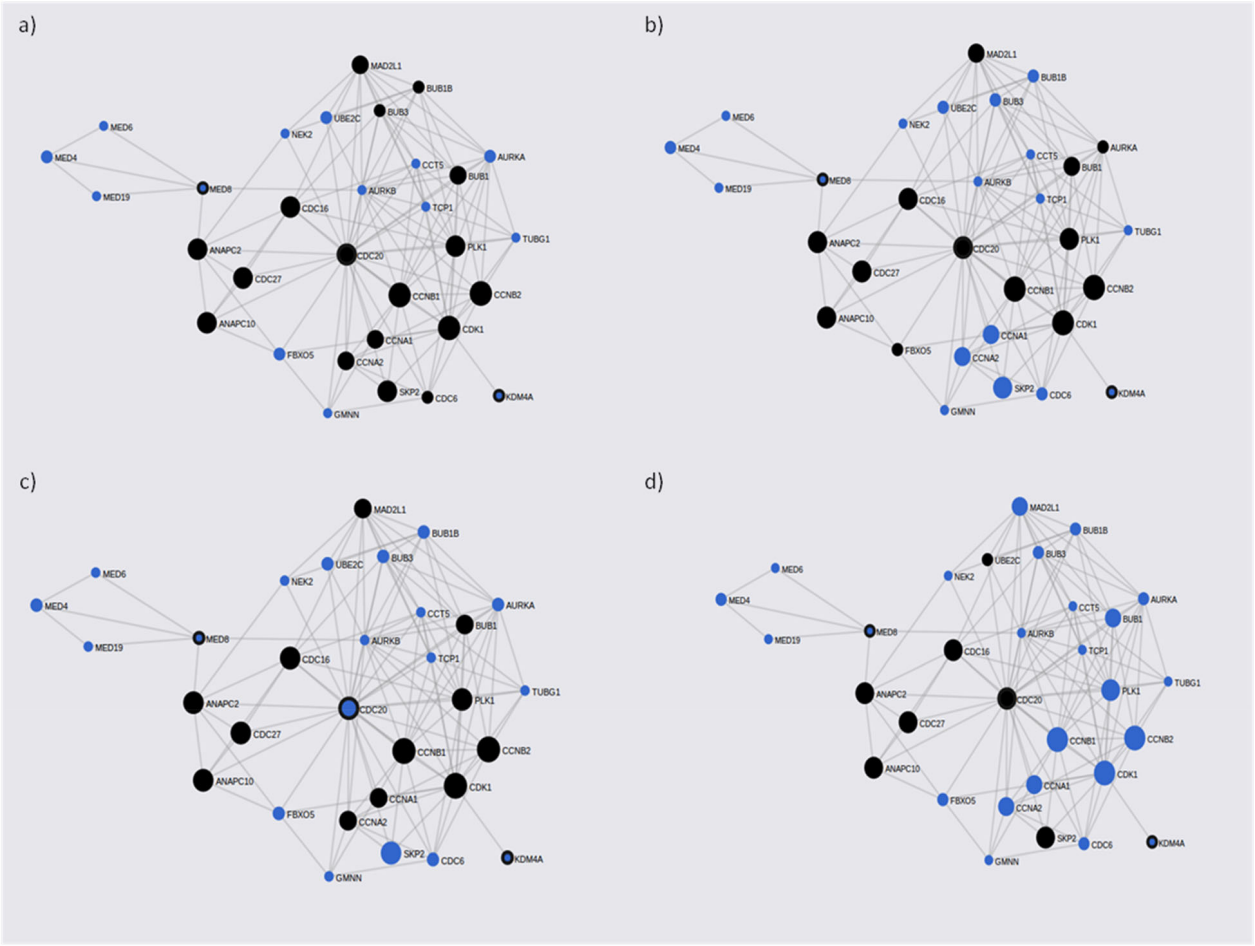
ADHD gene-networks constructed with PASCAL associated genes and its FunCoup interactors. Main query and interactor partners which form each network are represented. as blue circles. Query genes are also circled by black lines. Node sizes scale to emphasize gene importance in the whole network while participating nodes for each KEGG pathway are marked in black: **a** cell cycle; **b** oocyte meiosis; **c** progesterone-mediated oocyte maturation; **d** Ubiquitin mediated proteolysis

FunCoup has detected several interactors for the associated loci (Additional file 2: Table S2). Gene network for these genes was constructed including 16 query genes, 30 subnetwork genes and creating 124 links (Table 4). Enrichment analysis of KEGG pathways has revealed association for cell cycle (q-value = 2.41 × 10^− 20^), oocyte meiosis (q-value = 1.08 × 10^− 13^) and progesterone-mediated oocyte maturation (q-value = 1.4 × 10^− 13^) among others (Fig. 3) (Additional file 3: Table S3). Top associated molecular GO terms were tranferase activity, enzyme and ribonucleotide binding (Additional file 3: Table S3).

**Table 4:** Funcoup interactors detected using PASCAL associated genes as input. Some of the PASCAL genes were no detected by Funcoup, mostly antisense RNA genes (bold font). Moreover, some of the associated PASCAL genes were not detected by FUMA (underlined genes). Those genes marked in bold and underlined were not detected by both tools

Fuma input for ADHD genes		Fuma input for female ADHD genes	
PASCAL genes	FunCoup interactors	PASCAL genes	FunCoup interactors
*KDM4A*	*MED4*	*JMJD8*	*UBE2N*
***KDM4A-AS1***	*MED6*	*NARFL*	*HSPA8*
*ST3GAL3*	*MED19*	*WDR24*	*UBE2D2*
*PTPRF*	*ANAPC2*	*FBXL16*	*PSMA3*
*SZT2*	*AURKB*	*METRN*	*VCP*
*DUSP6*	*CERS2*	*FAM173A*	*SOD1*
*HYI*	*ELOVL2*	*CCDC78*	*CCT2*
*CDC20*	*MAD2L1*	*HAGHL*	*TCP1*
*ELOVL1*	*CCNB1*	*STUB1*	*CCT7*
*MED8*	*AURKA*		*PPP2R1A*
*MPL*	*BUB1B*		*CCT4*
*TIE1*	*PLK1*		*ILK*
*FEZF1*	*CCNA2*		*CCT5*
*SORCS3*	*UBE2C*		*HSP90AB1*
***FEZF1-AS1***	*CDK1*		*TUBA1B*
*CDH8*	*NEK2*		*MIF*
*SEMA6D*	*BUB1*		*PSMC5*
***NS3BP***	*CCNB2*		*UBE2V2*
*PDDC1*	*CCT5*		*TUBB*
	*BUB3*		*PSMD3*
	*ANAPC10*		*CCT8*
	*CDC6*		*PHB*
	*FBXO5*		*PA2G4*
	*SKP2*		*TUBA1A*
	*CDC27*		*UBE2D3*
	*GMNN*		*UBE2D1*
	*CCNA1*		*CIAO1*
	*TUBG1*		*FAM96B*
	*CDC16*		
	*TCP1*

**Fig. 4:**
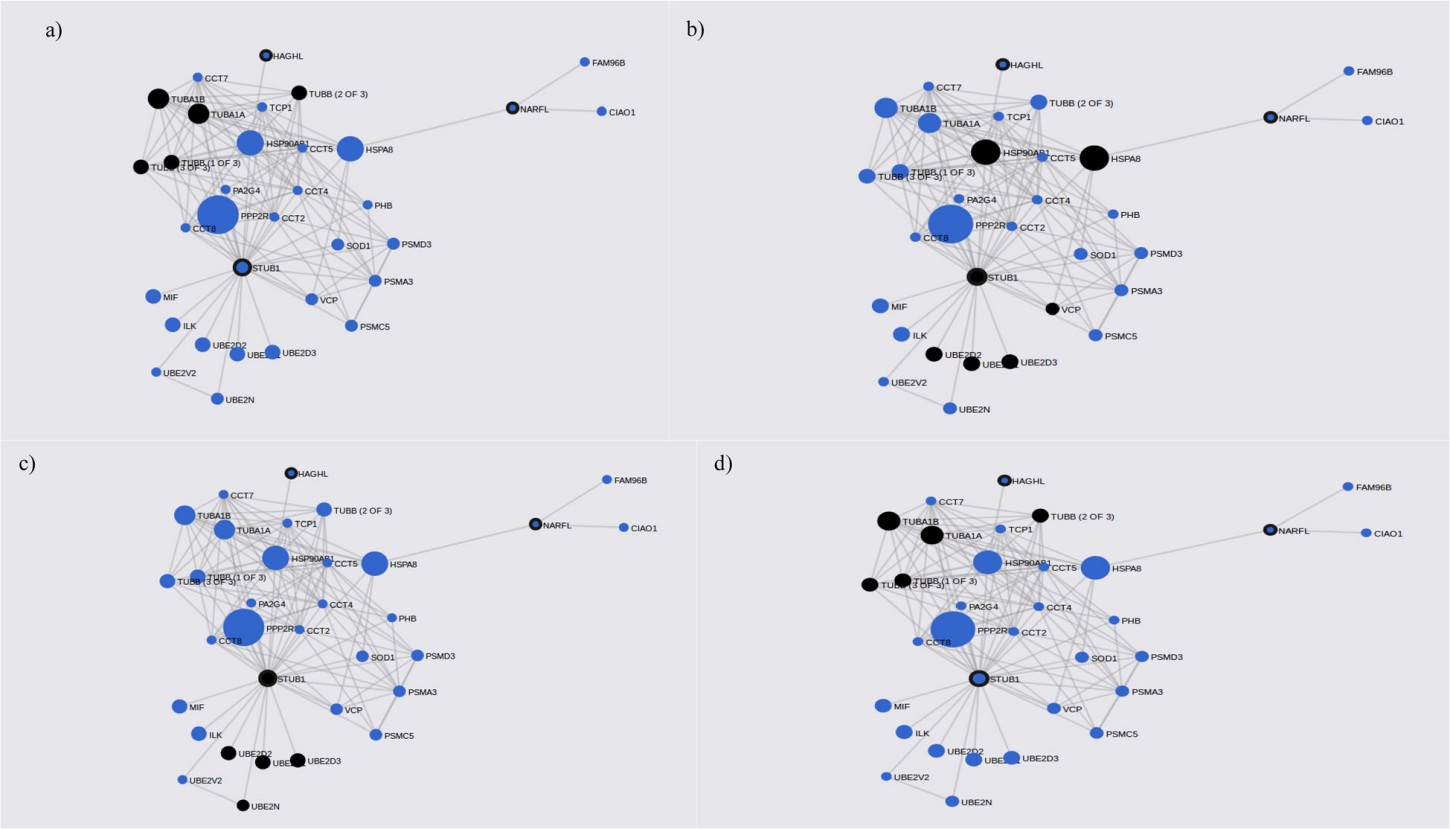
ADHD female gene-networks constructed with PASCAL associated genes and its FunCoup interactors. Mainquery and interactor partners which form each network are represented. as blue circles. Query genes are also circled by black lines. Node sizes scale to emphasize gene importance in the whole network while participating nodes for each KEGG pathway are marked in black: **a** GAP junction; **b** Protein processing in endoplasmic reticulum: **c** Ubiquitin mediated proteolysis; **d** Phagosome

The gene-network constructed with genes from the female subgroup includes 37 genes (9 query genes plus 28 subnetwork genes) (Table 4). Enrichment analysis of KEGG and GO terms has revealed significant results for GAP junction (q-value = 2.65 × 10^− 5^) and ribonucleotide binding (q-value = 2.03 × 10^− 9^) among others (Fig. 4) (Additional file 4: Table S4).

### Functional annotation

#### -Gene expression heatmaps and differentially expressed gene analysis (DEG)

**Fig. 5:**
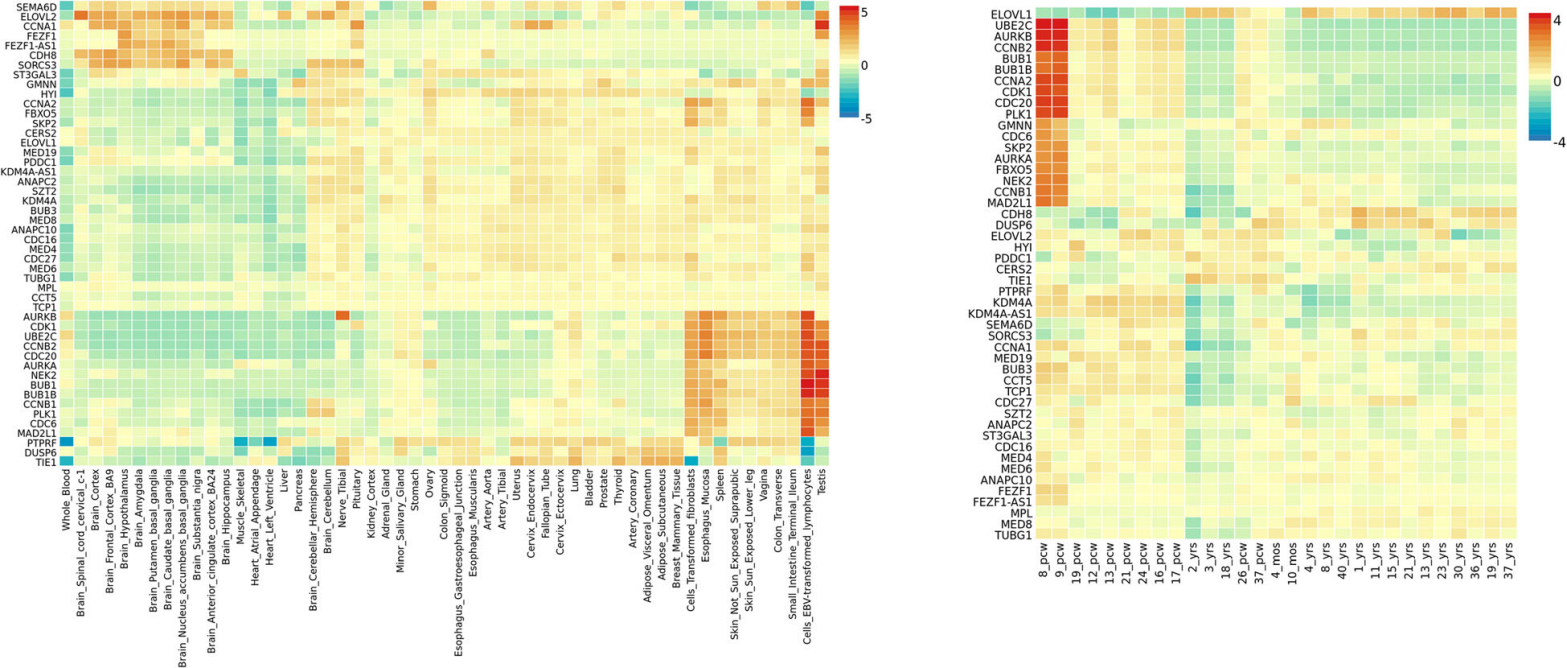
ADHD gene expression heatmaps constructed with GTEX v7 (53 tissues) (left) and BrainSpan 29 different ages of brain samples data (right).Genes and tissues are ordered by clusters for the GTEX heatmap. In the case of BrainSpan heatmap, genes are ordered by expression clusters and neurodevelopmental stages are chronologically ordered fom prenatal to postnatal stages

**Fig. 6:**
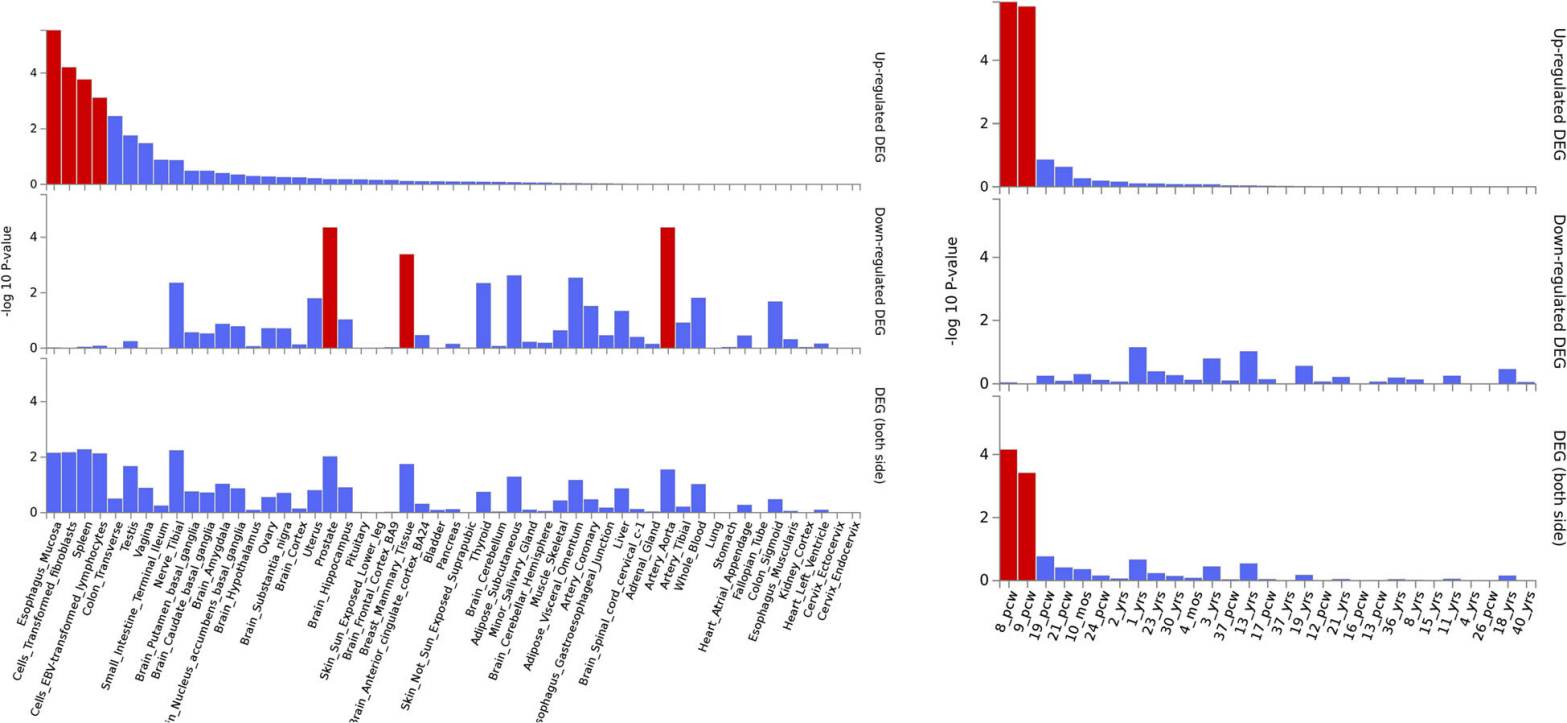
ADHD DEG plots constructed with GTEX v7 (53 tissues) (left) and BrainSpan 29 different ages of brain samples RNA seq data (right). Significantly enriched DEG sets (Pbon < 0.05) are highlighted in red

ADHD gene expression heatmap based on GTEX v7 RNA-seq data for 48 genes (18 PASCAL associated genes plus 30 interactor genes) has revealed higher relative expression levels across several brain tissues for the following genes: *ELOVL2, CCNA1, FEZF1, FEZF1-AS1, CDH8* and *SORCS3*. Conversely, the vast majority of the remaining genes, including those associated on chromosome 1, have shown relative lower expression levels in brain tissues from GTEX (Fig. 5). Expression heatmap based on BrainSpan data has not revealed any different expression between prenatal and postnatal stages for these genes. However, it is noticeable that a second cluster of genes demonstrated higher relative expression levels during early prenatal stages (8–9 postconception weeks (pcw)) in comparison with postnatal stages (*UBE2C, AURKB, CCN2B, BUB1, BUB1B, CCNA2, CDK1, CDC20, PLK1, GMNN, CDC6, SKP2, AURKA, FBX05, NEK2, CCNB1, MAD2L1)* (Fig. 5). Differentially Expressed Gene (DEG) analyses for ADHD data in several human tissues show significant up-regulation across esophagus, cell transformed fibroblasts, lymphocytes and spleen but not in any brain tissue. DEG graphs containing BrainSpan information have revealed significant upregulation during 8–9 pcw (early prenatal stages), which strongly correlates with the gene expression heatmap for the second cluster of genes (Fig. 6).

**Fig. 7:**
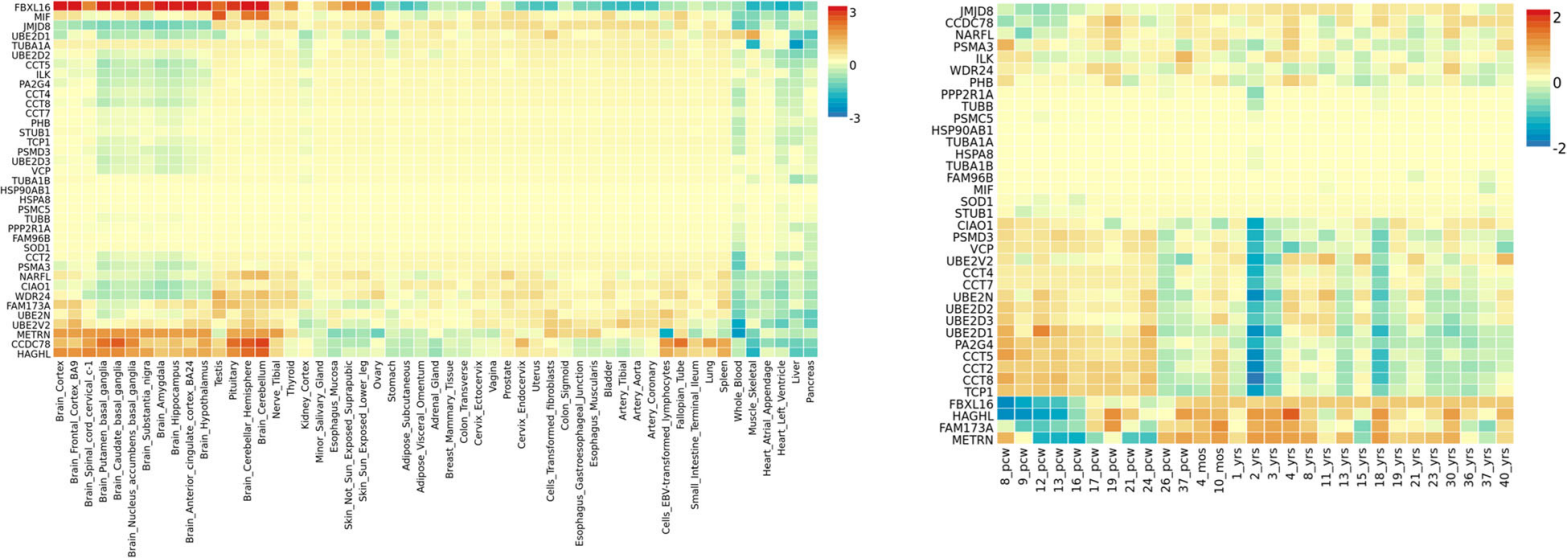
Gene expression heatmaps for ADHD females genes, constructed with GTEX v7 (53 tissues) (left) and BrainSpan 29 different ages of brain samples data (right).Genes and tissues are ordered by clusters for the GTEX heatmap. In the case of BrainSpan heatmap, genes are ordered by expression clusters and neurodevelopmental stages are chronologically ordered fom prenatal to postnatal stages

**Fig. 8:**
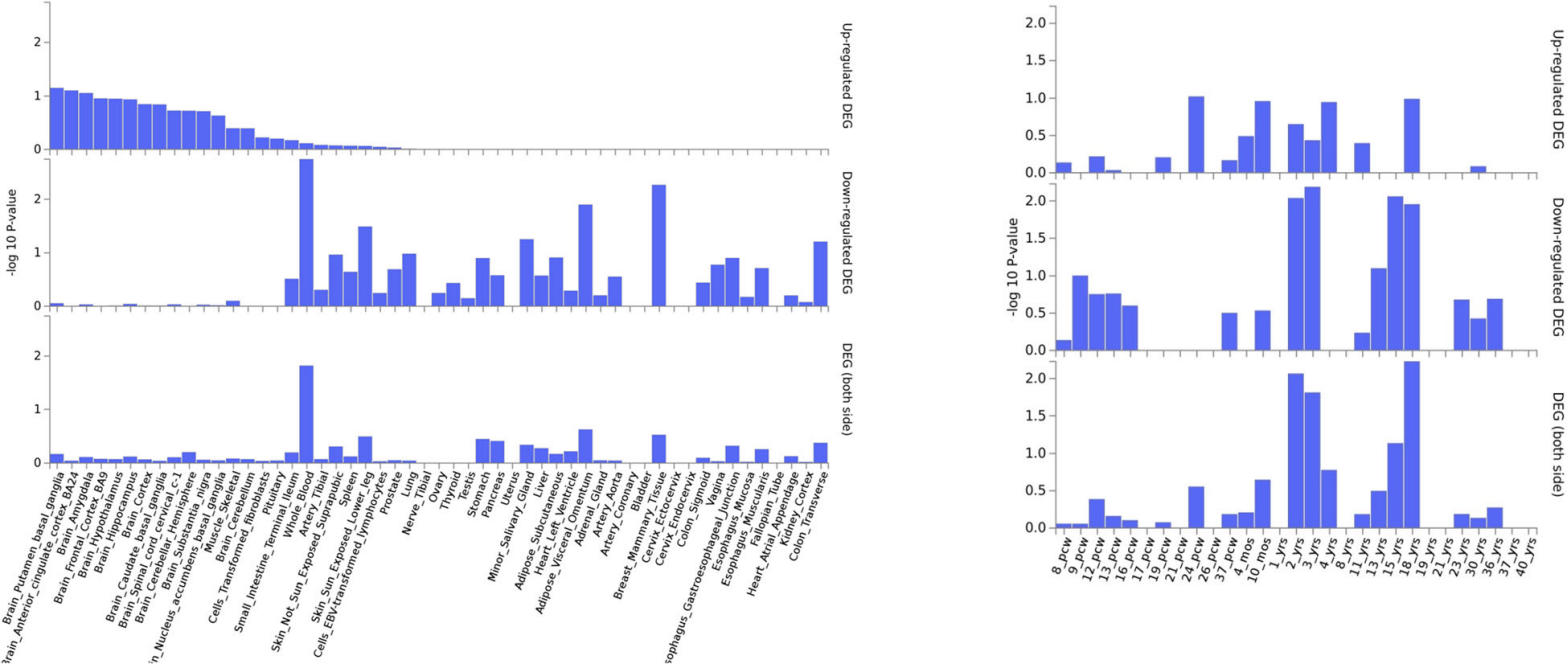
ADHD DEG plots for the females group constructed with GTEX v7 (53 tissues) (left) and BrainSpan 29 different ages of brain samples. No significantly enriched DEG set was found

ADHD expression heatmap for the female subgroup (9 query genes plus 28 interactor genes) has outlined *FBXL16* as the top up-regulated gene when relative expression levels were analyzed across the brain tissues included in GTEX but also in testis. Moreover, a second gene cluster including *METRN, CCDC78* and *HAGHL* has also shown higher relative expression levels across the same tissues that *FBXL16*. BrainSpan heatmap has demonstrated that *FBXL16* is downregulated during early and mid-prenatal stages (8,9,12,13 pcw) starting to progressively increase its expression from this stage onwards. *METRN* and *HAGHL* have also shown a similar trend (Fig. 7). Moreover, another cluster of genes that have shown high relative expression levels during prenatal stages (*UBE2D1, CCT2, CCT5, CCT8, PA2G4)* but it also has exhibited the opposite trend during the first stages of development followed by a variable expression pattern. However, DEG analyses have not shown any significant result (Fig. 8).

## Discussion

PASCAL algorithm has revealed novel ADHD associated genes ADHD in comparison with those genes previously reported by MAGMA [10]. The fact that both GBA methods employ similar but slightly different statistical approaches, might explain the differences found between the results reported by both methods. Thus, the vast majority of genes shared between both studies are located near to genome-wide significant index variants identified by Demontis et al. (cluster of genes within chromosome 1).

However PASCAL has been able to unveil new associated genes that do not physically overlap with individual genome wide significant SNPs. This is the case of the region located on chromosome 7 that encompass *FEZF1*, *FEZF1-AS1* and part of *CADPS2* including its *3’UTR*. The top SNP, rs2845270, is located between the *3’UTR* of *CADPS2* and just before the TSS (*transcription starting site*) of *FEZF1*. It should be noted that rs2845270 did not reach the required GWAS significance level (*p*-value < 5 × 10^− 8^). However, PASCAL algorithm was able to rescue *FEZF1* and *FEZF1-AS1* as associated genes when the *p*-values of neighbor SNPs were considered (all SNPs included between both recombination peaks). It is worth noting that the enrichment analysis has not detected any interactor for *FEZF1.* In addition*, FEZF1-AS1*, which encodes a lncRNA, was not recognized by FunCoup. This implies a limitation when it comes to describe the biological processes that could be underlying ADHD etiology in relation with this gene. Moreover, expression heatmaps have not revealed differences when *FEZF1* and *FEZF1-AS1* expression patterns were analyzed across pre- and post-natal developmental stages. However, GTEX expression analyses have identified two ADHD expression clusters (one downregulated and another one upregulated). Thus, *FEZF1* and *FEZF1-AS1* have shown a high relative expression in brain together with other ADHD associated genes and their interactors *(ELOVL2, CCNA1,CHD8* and *SORCS3)*. It should be noted that previous genetic and functional studies have linked this genomic region to other neurodevelopmental phenotypes. Therefore, *FEZF1* was identified as a strong candidate gene for ASD in a family sequencing study and the region spans the autism susceptibly locus 1 (AUTS1) [16, 17]. Moreover, *FEZF1* expression was mainly found in the forebrain region during early embriogenesis. *FEZF1* and *FEZF2* are both related with the differentiation of neuron stem cells and a proper cortical development [18–20]. In addition it was proved that the downregulation of lncRNA *FEZF1-AS1,* suppresses the activation of the Wnt/β-catenin signaling pathway in tumor progression. Although its functional role in neurons has not been proved to date, genes within this canonical pathway has been repeatedly linked to ASD and ADHD phenotypes [21, 22]. Moreover, *CADPS2* plays an important function on the synaptic circuits throughout the activity-dependent release of neurotrophic factor (BDNF). Indeed, genetic variants in *CADPS2* gene were associated to ASD and *CADPS2* KO mice have shown impairments in behavioral phenotypes [23–26].

In addition, two novel loci were associated with ADHD, *NS3BP* and *PDDC1,* both located on chromosome 11. Previous GBA carried out by MAGMA has revealed association of a nearby locus, *PIDD1,* but the proxy SNP was not identified since the required GWAS significance level (*p* < 5 × 10 ^− 8^) was not surpassed [10]. However, PASCAL was able to identify as associated both genes located on the same genetic region around the top SNP (rs28633403). FunCoup was unable to recognize *NS3BP* which belongs to an uncharacterized *LOC171391* gene which entails a limitation in the enrichment analysis. Moreover, no gene interaction was reported for *PDDC1*. Thus, it should be noted that it has not been possible to directly relate *PDDC1* (glutamine amidotransferase like class 1 domain containing 1) and *NS3BP* (pseudogene) with any biological term. Moreover, there is a lack of previous studies reporting their biological function. However, *PDDC1* is located within the brain-downregulated cluster together with other ADHD associated genes and their interactors (*KDM4A, KDM4A-AS1, CDC20, AURKA, NEK2, BUB1* and *BUB1B* among others). Curiously, some of the genes that have shown downregulation across GTEX adult brain were upregulated during early neurodevelopmental stages (8–9 pcw) and they have shown a high relative expression in testis. Precisely, most of those genes overexpressed in testis are interactor partners of *CDC20*. *CDC20* together with *KDM4A* and *KDM4A-AS1* has shown enrichment in KEGG and GO terms related with cell cycle as well as with positive and negative regulation of cellular processes. Specifically, *CDC20* has been previously reported as a coactivator of the ubiquitin ligase anaphase-promoting complex (APC). The APC-CDC20 complex has essential functions in regulating mitosis but it has also been described nonmitotic functions in neurons. Thus, APC-CDC20 complex plays a role in dendrite morphogenesis during brain development [27].

Novel associated genes were reported in both males and females when PASCAL analysis was carried out. Until now, no GBA has been done with this data. However, a genetic investigation of sex bias in ADHD including this GWAS meta-analyses has revealed different single SNPs associated for male and female meta-analyses [28]. Thus, PASCAL has revealed the association of 9 genes located on chromosome 16. The lead SNP of the region (rs4984677) lies within *FBXL16* and it has been pointed as one of the top SNPs associated (*p*-value:1.9 × 10^− 7^) for females in the sex-specific meta-analysis [28]. However, it should be noted that the number of SNPs covering these genes is relatively lower in comparison with other associated genes. Moreover, it is also necessary to highlight that the female cohort only includes 4945 cases versus 14,154 cases included in the male cohort. Therefore, these associations should be carefully considered.

Network analysis for the whole GBA, has detected 9 associated genes but it was only able to identify interactors for three genes: *NARFL, HAGHL* and *STUB.* In addition, enrichment analyses seem to point to different biological processes from those previously reported for the whole ADHD analysis. Gene expression heatmap (GTEx data) has shown a higher expression in brain for *FBXL16, MTRN, CCDC78* and *HAGHL* compared to other tissue types. It is worth noting that the expression of *FBXL16, HAGHL* and *MTRN* cluster together across different neurodevelopmental stages (prenatal and postnatal). The function of these genes during neurodevelopment is unknown except in the case of *MTRN. MTRN e*ncodes for a neurotrophic factor that plays important roles both in the glial cell differentiation and the formation of axonal networks [29].

The genetic and functional annotation results seem to point to a differential role of the associated genes in males versus females. Genetic differences between genders in ADHD etiology could explain the sex bias reported in the prevalence for this NDD. Thus, males have shown a rate of ADHD diagnosis seven times higher than females [30]. However, the study conducted by the PGC did not found any evidence that point towards the explanation of this sex bias by the association of common variants [28]. Further research will be needed to clarify this question. Probably, to gather a much larger sample size for males and females GWAS would be helpful for future studies.

In conclusion, PASCAL algorithm was used to carry out a novel ADHD GBA, employing summary statistics from the latest PGC meta-analysis. This has helped to identify novel gene associations for ADHD different from those reported by MAGMA. Thus, our results prove that both tools might be used as complementary GBA approaches to highlight genes associated to this disorder.

Although PASCAL has solved many limitations found in other GBA as the algorithmic efficiency and the optimization of the correlation matrix, novel improvements could be added to the method. Thus, the incorporation of functional annotation data, eQTLs or methylation status for each SNP could help to prioritize and report different associated genes.

Moreover, gene-network and functional annotation approaches including gene expression heatmaps and DEG have helped to understand these genetic findings in a biological context. This is extremely useful to select the most suitable candidates genes for future functional studies.

## Methods

GWAS meta-analysis (ADHD) GWAS summary statistics from the latest ADHD GWAS meta-analysis were obtained from the public repository available in the PGC website (https://www.med.unc.edu/pgc/results-and-downloads/). The PGC’s policy (https://www.med.unc.edu/pgc) is to make genome-wide summary results public. Summary statistics from the following files were employed as input for our analysis: (adhd_eur_jun2017.gz, META_PGC_iPSYCH_males.gz, and META_PGC_iPSYCH_females.gz). These ADHD GWAS meta-analysis (full European ADHD GWAS and separated by sex) were also retrieved from the PGC and iPSYCH analysis released in June 2017 (hg19).

In the case of the ADHD male data set, 14,154 cases and 17,948 controls were considered. However, ADHD female dataset only include 4945 cases and 16,246 controls. Additional data about each individual GWAS included in each meta-analysis data set including sample size, ancestry and which diagnostic instrument was employed can be found in Additional file 1: Table S1. Additional information about the genotyping and QC (quality control) methods and the summary statistics employed can be found at PGC website (http://www.med.unc.edu/pgc/results-and-downloads).

### Gene-based analysis (GBA)

GBA was carried out using PASCAL (https://www2.unil.ch/cbg/index.php?title=Pascal). It has employed as an input the summary statistics obtained from the ADHD meta-analysis. Individual SNPs from GWAS results were first mapped to genes employing a default ±50 kb window around the start and gene end. The default maximum number of SNPs per gene allowed by PASCAL was 3000. Moreover, LD information from 1000 Genomes was employed by PASCAL in order to consider linkage between markers for each gene. The Bonferroni correction sets the significance cut-off at 2.26 × 10^− 6^ (0.05 / 22,135 genes). This number of genes includes the whole UCSC list (hg19) employed by PASCAL to perform calculations. However, for each ADHD data set the number of genes considered in each individual analysis is slightly lower. However, it was preferred to consider the most conservative threshold. The whole list of gene scores calculated by PASCAL for each ADHD data set can be consulted in the electronic (Additional files 5, 6, 7).

### Regional plots

LocusZoom tool (http://locuszoom.org/) was employed to construct regional plots for those genetic regions containing PASCAL associated genes. To this aim, meta-analysis data including marker name, *p*-values, OR, chromosome position (start-end) and index SNP, were specified for each one of the corresponding studies (ADHD whole, ADHD females and males). The source of LD information used to construct the r^2^ correlation between SNPs in these regional plots was retrieved from hg19/1000 Genomes Nov 2014 EUR (Europe). The rest of optional controls were used as default.

### Gene-network analysis

FunCoup v.4.0 (http://funcoup.sbc.su.se/search/) was employed to expand the associated list of genes (*p* < 2.26 × 10^− 6^) previously obtained and to include its interactors. This database integrates 10 different types of functional couplings among genes that allow to infer functional association networks: Protein interaction (PIN), Mrna Co-expression (MEX), Protein Co-expression (PEX), Genetic Interaction profile similarity (GIN), Shared Transcription factor binding (TFB), Co-miRNA regulation by shared miRNA targeting (MIR), Subcelular Co-localization (SCL), Domain interactions (DOM), Phylogenetic profile simarity (PHP) and Quantitative mass spectrometry (QMS). Gene networks for the ADHD whole analysis were constructed considering as input 16 of the 19 associated genes because 3 of them were not recognized by the tool (Table 4). Gene networks were constructed considering three different parameters. Therefore, expansion parameters include: confidence threshold (0.8), a maximum number of 30 nodes per expansion step and a query depth of 1 (only genes directly linked to the query genes are shown). Network expansion algorithm was settled in order to obtain those strongest interactors for any query gene, without prioritizing common neighbor’s links. Moreover, enriched term analysis (KEGG and GO molecular function) were considered for each gene-network constructed with the corresponding *p*-values. Gene-network representation displays the most significant KEGG pathways according to their q-values after considering an FDR approach. Node sizes scale to emphasize gene importance in the whole network while participating nodes for each KEGG pathway are marked in black.

### Functional annotation

GENE2FUNC, a core process of FUMA (*Functional Mapping and Annotation of Genome-Wide Association Studies)* (http://fuma.ctglab.nl/), was employed to functional annotate associated genes and its interactors. In the case of ADHD, a set of 48 genes was used as input (18 PASCAL associated genes plus 30 strong interactors from FunCoup). Moreover, for the female’s subset, a list of 37 genes was employed (9 PASCAL associated genes plus 28 FunCoup interactors) (Table 4).

Different analyses performed by GENE2FUNC were employed, including a gene expression heatmap and enrichment of differentially expressed genes (DEG). Gene expression heatmap was constructed employing GTEx v7 (53 tissue types) and BrainSpan RNA-seq data. The average of normalized expression per label (zero means across samples) was displayed on the corresponding heatmaps. Expression values are TPM (Transcripts Per Million) for GTEx v7 and RPKM (Read Per Kilobase per Million) in the case of BrainSpan data set. Heatmaps display normalized expression value (zero mean normalization of log2 transformed expression) and darker red means higher relative expression of that gene in each label, compared to a darker blue color in the same label. DEG analysis was carried out creating differentially expressed genes for each expression data set. In order to define DEG sets, two-sided Student’s t-test were performed per gene per tissue against the remaining labels (tissue types or developmental stages). Those genes with a *p*-value < 0.05 after Bonferroni correction and a log fold change ≥0.58 are defined as DEG. The direction of expression was considered. The -log10 (*p*-value) refers to the probability of the hypergeometric test.

## Supplementary information

**Additional file 1: Table S1.** Characterization of ADHD cohorts included in the PGC GWAS metanalyses.

**Additional file 2: Table S2.** Query genes and interactors detected by Funcoup for the loci associated for PASCAL GBA in the global analysis and female analysis.

**Additional file 3: Table S3.** Enriched terms for query ADHD genes and its interactors (subnetwork genes) according to Funcoup.

**Additional file 4: Table S4.** Enriched terms for query genes in the ADHD females subgroup and its interactors (subnetwork genes) according to Funcoup.

**Additional file 5.** adhd_eur_jun2017.genebased.sum.genescores.xls.

**Additional file 6.** GWASPGCfemalesadhdpvalor.sum.genescores.xls.

**Additional file 7. **GWASPGCmalesadhdpvalor.sum.genescores.xls. For each file different columns are represented: chromosome, start-end positions, strand (+ or -) relative to transcription start, gene symbol (UCSC), numSNPs (number of SNPs employed to calculate the gene score), *p*-value calculated by PASCAL.

## Data Availability

All data generated during this study are included in this published article and its Additional files. Summary statistics for each ADHD GWAS are publicly available at: https://www.med.unc.edu/pgc/results-and-downloads.

## Abbreviations

DEG: Differentially Expressed Gene
ADHD: Attention Deficit Hyperactiviy Disorder
ASD: Autism Spectrum Disorder
BDNF: Neurotrophic factor
CNV: Copy number variation
DOM: Domain interactions
FDR: False discovery rate
FUMA: Functional Mapping and Annotation of Genome-Wide Association Studies
GBA: Gene-based analysis
GIN: Genetic Interaction profile similarity
GO: Gene ontology
GTEx: Genotype-tissue expression
GWAS: Genome-Wide Association Study
IPSYCH: Lundbeck Foundation Initiative for Integrative Psychiatric Research
KEGG: Kyoto Encyclopedia of Genes and Genomes
LD: Linkage disequilibrium
MEX: mRNA co-expression
MIR: Co-miRNA regulation by shared miRNA targeting
NDD: Neurodevelopmental disorder
OR: Odds ratio
PASCAL: Pathway Scoring Algorithm
PEX: Protein Co-expression
PGC: Psychiatric genomic consortium
PHP: Phylogenetic profile similarity
PIN: Protein interaction
QC: Quality control
QMS: Quantitative mass spectrometry
RPKM: Read per kilobase per million
SCL: Subcelular Co-localization
SNP: Single nucleotide polymorphism
TFB: Shared Transcription factor binding
TPM: Transcripts per million
TSS: Transcription starting site
VEGAS: Versatile gene-based association study
