# Supplementary material for: Gene-based analysis of ADHD using PASCAL: a biological insight into the novel associated genes
Source: BMC Med Genomics. 2019 Oct 24;12:143. doi: 10.1186/s12920-019-0593-5 (PMC6813133; doi:10.1186/s12920-019-0593-5)
Supplement: Supplementary file 1 — Additional file 1: Table S1. Characterization of ADHD cohorts included in the PGC GWAS metanalyses. [file 12920_2019_593_MOESM1_ESM.docx]

| Phenotype | Study name | | Design study | Sample size (cases/controls) | Ancestry | Diagnostic instrument | Total (cases/controls) |
| --- | --- | --- | --- | --- | --- | --- | --- |
| ADHD | iPSYCH | | Case/Control | 14584/22492 | European | ICD10 criteria | 19099/34197  Males only  14154/17948  Females only  4945/16246 |
|  | PGC | Children´s Hospital Philadelphia (CHOP) | Trio | 262/262 | European | K-SADS |  |
|  |  | IMAGE-I | Trio | 700/700 | European | PACS, Conners ADHD rating scales, SDQ |  |
|  |  | PUWMa, USA | Trio | 563/563 | European | K-SADS-E, MAGIC, DSM-V criteria, K-SADS-PL, SADS-LA-IV, SNAP-IV, CBCL |  |
|  |  | Toronto | Trio | 109/109 | European | PICS, TTI |  |
|  |  | Barcelona | Case/Control | 572/425 | European | DSM-V criteria, CAADID, CGI |  |
|  |  | Bergen, Norway | Case/Control | 295/202 | European | Norwegian National Medical Registry |  |
|  |  | Cardiff, UK | Case/Control | 721/5081 | European | CAPA |  |
|  |  | Germany | Case/Control | 487/1290 | European | KSADS-PL, FBB-MKS |  |
|  |  | IMAGE-II | Case/Control | 624/1755 | European | (Kiddie-Sads-PL-German Version or Kinder-DIPS  (DISC-P), Conners Questionnaires, CBCL and TRF |  |
|  |  | Yale Penn | Case/Control | 182/1315 | European | DSM-V Criteria |  |

**Table** 1**. Characterization of ADHD cohorts included in the PGC GWAS metanalyses.**

Abbreviations: ICD10: International Classification of Diseases, 10^th^ revision; K-SADS: Schedule for Affective Disorders and Schizophrenia for School-Age Children;K-SADS-E: Schedule for Affective Disorders and Schizophrenia for School-Age Children, Epidemiologic Version; PACS: Parental Account of Childhood Symptom; SDQ: Strengths and Difficulties Questionnaire; MAGIC: Missouri Assessment of Genetics Interview for Children; K-SADS-PL: Schedule for Affective Disorders and Schizophrenia for School-Age Children, Present and Lifetime version; SADS-LA-IV: Lifetime version; SNAP-IV: parent and teacher versions of the Swanson, Nolan, and Pelham, version IV; CBCL: parent-completed Childhood Behavior Checklist; TRF: Teacher Report Form; PICS : Parent Interview for Child Symptoms; TTI: Teacher Telephone Interview; CAADID: Conner’s Adult ADHD Diagnostic Interview for DSM-IV; CGI: Impairment was measured with the Clinical Global Impression; CAPA: Child and Adolescent Psychiatric 11 Assessment, Parent Version; FBB-HKS: German teacher rating scale for ADHD; DSM-V
