# Supplementary material for: Gene-based analysis of ADHD using PASCAL: a biological insight into the novel associated genes
Source: BMC Med Genomics. 2019 Oct 24;12:143. doi: 10.1186/s12920-019-0593-5 (PMC6813133; doi:10.1186/s12920-019-0593-5)
Supplement: Supplementary file 2 — Additional file 2: Table S2. Query genes and interactors detected by Funcoup for the loci associated for PASCAL GBA in the global analysis and female analysis. [file 12920_2019_593_MOESM2_ESM.docx]

| P**ASCAL analysis** | **Query genes** | **Interaction partners** | **Network** |
| --- | --- | --- | --- |
| **ADHD** | *CDC20* | *MAD2L1,CCNB1,AURKB,AURKA,BUB1B,PLK1,CCNA2,UBE2C,CDK1,NEK2,BUB1,CCNB2,*  *CCT5,BUB3,ANAPC10,CDC6,ANAPC2,FBXO5,SKP2,CDC27,GMNN,CCNA1,TUBG1,CDC16,*  *TPC1* | Complex and PPI |
|  | *MED8* | *MED4,MED6,MEED19,ANAPC2,AURKB* | Complex and PPI |
|  | *ELOVL1* | *CERS2,ELOVL2* | PPI |
|  | *KDM4A* | *CDK1* | PPI |
| **ADHD females** | *STUB1* | *UBE2N,HSPA8,UBE2D2,PSMA3,VCP,SOD1,CCT2,TCP1,CCT7,PPP2R1A,CCT4,ILK,CCT5,*  *HSP90AB1,TUBA1B,MIF,PSMC5,UBE2V2,TUBB,PSMD3,CCT8,PHB,PA2G4,TUBA1A,UBE2D3,*  *UBE2D1* | Complex and PPI |
|  | *NARFL1* | *XH**S**P90AB1* | PPI |
|  | *HAGHL* | *CIAO1,FAM96B,HSPA8* | PPI |

**Table 2. Query genes and interactors detected by Funcoup for the loci associated for PASCAL GBA in the global analysis and female analysis.** Only those genes for which interactors were found are shown in the table. Network column indicates the type of functional coupling among genes
