# Supplementary material for: Gene-based analysis of ADHD using PASCAL: a biological insight into the novel associated genes
Source: BMC Med Genomics. 2019 Oct 24;12:143. doi: 10.1186/s12920-019-0593-5 (PMC6813133; doi:10.1186/s12920-019-0593-5)
Supplement: Supplementary file 3 — Additional file 3: Table S3. Enriched terms for query ADHD genes and its interactors (subnetwork genes) according to Funcoup. [file 12920_2019_593_MOESM3_ESM.docx]

| **Enriched terms** |  | **Genes** | **q value** |
| --- | --- | --- | --- |
| KEEG signaling | Cell cycle | *CDC20,CDK1,BUB1,BUB3,,MAD2L1,SKP2,ANAPC2,CCNB1,,BUB1B,CCNB2,CDC27,PLK1,CCNA2,ANAPC10,CDC16,CCNA1,CDC6,* | 2.41 x 10^-20^ |
|  | Oocyte meiosis | *CDK1,CDC20,BUB3,AURKA,MAD2L1,ANAPC2,,CCNB1,CCNB2,CDC27,PLK1,FBXO5,ANAPC10,CDC16,* | 1.08 x 10^-13^ |
|  | Progesterone-mediated oocyte maturation | *CDK1,BUB1,MAD2L1,ANAPC2,CCNB1,CCNB2,CDC27,PLK1,CCNA2,ANAPC10,CDC16,CCNA1* | 1.4 x 10^-13^ |
| KEEG metabolic | Ubiquitin mediated proteolysis | *CDC20,SKP2,ANAPC2,UBE2C,CDC27,ANAPC10,CDC16* | 3.15 x 10^-7^ |
| Go molecular function | Tranferase activity | *ST3GAL3,TIE1,ELOVL1,CDK1,BUB1,AURKA,SKP2,NEK2,ANAPC2,UBE2C,AURKB,CCNB1,*  *BUB1B,CCNB2,PLK1,CCNA2,* | 8.55 x 10^-4^ |
|  | transferase activity, transferring phosphorus-containing groups | *TIE1,CDK1,BUB1,BUB3,AURKA,NEK2,AURKB,CCNB1,BUB1B,CCNB2,PLK1,CCNA2* | 1.17 x 10^-3^ |
|  | enzyme binding | *KDM4A,CDC20,AURKA,NEK2,ANAPC2,UBE2C,CCNB1,TCP1,CDC27,PLK1,CCNA2,FBXO5,*  *GMNN,CDC6* | 3.69 x 10^-3^ |
|  | ribonucleotide binding | *TIE1,CDK1,CCT5,BUB1,AURKA,TUBG1,NEK2,UBE2C,AURKB,TCP1,BUB1B,PLK1,CDC6* | 1.12 x 10^-2^ |
|  | carbohydrate derivative binding | *PTPRF,TIE1,CDK1,CCT5,BUB1,AURKA,TUBG1,NEK2,UBE2C,AURKB,TCP1,BUB1B,PLK1,CDC6* | 1.39 x 10^-2^ |
|  | nucleotide binding | *TIE1,CDK1,CCT5,BUB1,AURKA,TUBG1,NEK2,UBE2C,AURKB,TCP1,BUB1B,PLK1,CDC6* | 1.82 x 10^-2^ |
|  | nucleoside phosphate binding | *TIE1,CDK1,CCT5,BUB1,AURKA,TUBG1,NEK2,UBE2C,AURKB,TCP1,BUB1B,PLK1,CDC6* | 1.82 x 10^-2^ |
|  | protein binding | *CDK1,CCT5,BUB1,BUB3,AURKA,MAD2L1,SKP2,TUBG1,NEK2,ANAPC2,MED19,UBE2C,AURKB,CCNB1,TCP1,BUB1B,CCNB2,CDC27,PLK1,CCNA2,MED6,FBXO5,GMNN,HYI,CDC16,CCNA1,*  *CDC6,MED4,ELOVL2,CERS2,MED8,KDM4A,PTPRF,TIE1,SEMA6D,ELOVL1,MPL,CDC20* | 1.82 x 10^-2^ |
|  | anion binding | *PTPRF,TIE1,CDK1,CCT5,BUB1,AURKA,TUBG1,NEK2,UBE2C,AURKB,TCP1,BUB1B,PLK1,*  *CDC6* | 4.1 x 10^-2^ |
|  | small molecule binding | *TIE1,CDK1,CCT5,BUB1,AURKA,TUBG1,NEK2,UBE2C,AURKB,TCP1,BUB1B,PLK1,CDC6* | 4.1 x 10^-2^ |
|  | transcription cofactor activity | *MED8,MED19,MED6,GMNN,MED4* | 4.1 x 10^-2^ |

**Table 3.Enriched terms for query ADHD genes and its interactors (subnetwork genes) according to Funcoup.**
