# Supplementary material for: Gene-based analysis of ADHD using PASCAL: a biological insight into the novel associated genes
Source: BMC Med Genomics. 2019 Oct 24;12:143. doi: 10.1186/s12920-019-0593-5 (PMC6813133; doi:10.1186/s12920-019-0593-5)
Supplement: Supplementary file 4 — Additional file 4: Table S4. Enriched terms for query genes in the ADHD females subgroup and its interactors (subnetwork genes) according to Funcoup. [file 12920_2019_593_MOESM4_ESM.docx]

| **Enriched terms** |  | **Genes** | **q value** |
| --- | --- | --- | --- |
| KEEG signaling | GAP junction | *TUBA1B,TUBB,TUBA1A* | 2.65 x 10^-5^ |
| KEEG metabolic | Protein processing in endoplasmic reticulum | *STUB1,UBE2D2,HSP90AB1,VCP,HSPA8,UBE2D1,UBE2D3* | 1.66 x 10^-4^ |
|  | Ubiquitin mediated proteolysis | *STUB1,UBE2D2,UBE2D1,UBE2N,UBE2D3* | 1.96 x 10^-3^ |
|  | Phagosome | *TUBA1B,TUBB ,TUBA1A* | 2.72 x 10^-2^ |
| GO molecular function | ribonucleotide binding | *UBE2D2,HSP90AB1,VCP,TUBA1B,HSPA8,PSMC5,TCP1,UBE2D1,CCT8,CCT4,TUBB,CCT2,SMA3,CCT7,UBE2N,,CCT5,UBE2D3,TUBA1A,ILK* | 2.03 x 10^-9^ |
|  | protein binding involved in protein folding | *TCP1,CCT8,CCT4,CCT2,CCT7,CCT5* | 2.03 x 10^-9^ |
|  | unfolded protein binding | *HSP90AB1,HSPA8,TCP1*  *CCT8,CCT4,CCT2,CCT7,CCT5* | 2.43 x 10^-9^ |
|  | nucleotide binding | *UBE2D2,HSP90AB1,VCP,TUBA1B,HSPA8,PSMC5,TCP1,UBE2D1,CCT8,CCT4,TUBB,CCT2,CCT7,UBE2N,CCT5,UBE2D3,TUBA1A,ILK* | 4.34 x 10^-9^ |
|  | nucleoside phosphate binding | *UBE2D2,HSP90AB1,VCP,TUBA1B,HSPA8,PSMC5,TCP1,UBE2D1,CCT8,CCT4,TUBB,CCT2,CCT7,UBE2N,CCT5,UBE2D3,TUBA1A,ILK* | 4.34 x 10^-9^ |
|  | carbohydrate derivative binding | *UBE2D2,HSP90AB1,VCP,TUBA1B,HSPA8,PSMC5,TCP1,UBE2D1,CCT8,CCT4,TUBB,CCT2,CCT7,UBE2N,CCT5,UBE2D3,TUBA1A,ILK* | 9.84 x 10^-9^ |
|  | small molecule binding | *UBE2D2,HSP90AB1,VCP,TUBA1B,HSPA8,PSMC5,TCP1,UBE2D1,CCT8,CCT4,TUBB,CCT2,CCT7,UBE2N,CCT5,UBE2D3,TUBA1A,ILK* | 2.22 x 10^-7^ |
|  | enzyme binding | *HSP90AB1,VCP,TUBA1B,HSPA8,TCP1,UBE2D1,TUBB,CCT2,PA2G4,CCT7,UBE2N,SOD1,PHB,ILK* | 1.26 x 10^-6^ |
|  | structural constituent of cytoskeleton | *TUBA1B,TUBB,TUBA1A* | 2.26 x 10^-5^ |
|  | protein domain specific binding | *STUB1,HSP90AB1,VCP,HSPA8,TUBB,TUBA1A,ILK* | 4.89 x 10^-5^ |

**Table 4. Enriched terms for query genes in the ADHD females subgroup and its interactors (subnetwork genes) according to Funcoup.**
